# Supplementary material for: Validation and implementation of a mobile app decision support system for prostate cancer to improve quality of tumor boards
Source: PLOS Digit Health. 2023 Jun 7;2(6):e0000054. doi: 10.1371/journal.pdig.0000054 (PMC10246794; doi:10.1371/journal.pdig.0000054)
Supplement: Attachment — Submitted filename: Response to Reviewers.pdf [file pdig.0000054.s001.pdf]

UNIKLINIK KÖLN
Klinik I für Innere Medizin
Onkologie, Hämatologie, Klinische Infektiologie, Klinische Immunologie,
Hämostaseologie und internistische Intensivmedizin

Centrum für Integrierte Onkologie Köln Bonn

Uniklinik Köln | Klinik I für Innere Medizin
Kerpener Str. 62 · 50937 Köln

PLOS Digital Health
Carlyle House, Carlyle Road
Cambridge, CB4 3DN
United Kingdom

Prof. Dr. med. Michael Hallek
Direktor der Klinik

Priv.-Doz. Dr. med. Thomas Elter
Facharzt für Innere Medizin,
Hämatologie und Onkologie

Telefon: (0221) 478-87714
Telefax: (0221) 478-97230
Email: thomas.elter@uk-koeln.de

Köln, 30.06.2022

Subject: **Rebuttal Letter**

Dear Prof. Dr. Narasimhan, Dear Oliver Ian Tolentino, Dear Reviewers,

Thank you very much for your critical review of our manuscript.
We would like to comment on the individual points of critic:

1. Framework or theory involved in the methods

The use and potential of expert-curated-decision support versus AI-based approaches for providing oncology treatment recommendations was described by the work of Bungartz et al in Nature Biotechnology[1].

For the purpose of good comparability, we considered it a legitimate approach to methodically use the preliminary work on Watson for Oncology as also published in PLOS One for our work. [2-21]

In particular, the work of Yu et al on the use of Watson for Oncology in 201 prostate cancer patients provided a useful comparison here using the same methods, noting the quantitatively superior analysis of 1873 patients in our work.[16]

2. Rationale for statistical analysis including appropriate statistical analyses/methods

Descriptive statistics and data analysis were carried out using IBM´s statistics software SPSS Version 25 and Microsoft Excel. The patient characteristics age, cancer stage, risk stratification, Gleason Score, and PSA-level (prostate specific antigen) were documented. Descriptive statistics were depicted as number of percentages or mean ± standard deviation (SD). After assigning patients to the concordant or the non-concordant group, a chi-squared test was used to compare categorical variables and the Mann-Whitney U test was applied to compare ordinal variables between the groups. Multivariate logistic regression analysis was used to analyze the association between the concordance rate and clinicopathological data. Statistical significance was assumed if the p-value was < 0.05 for all statistical analysis. Graphics, charts and tables were generated using SPSS, Microsoft Excel and Power Point.

3. Rationale for the study design and why a design like RCT wasn't chosen

The reason for the study rationale is provided in point 1 described above.
Randomization was not useful given that the data were collected retrospectively. Of course, the respective pairs (App and tumor board) of each treatment decision were blinded for the examination of concordance to avoid investigator bias.
However, the current work should serve as a basis to enable a prospective evaluation.
Currently, the technical implementation of supporting query algorithms is taking place through an API into existing hospital information systems, which will enable automatic therapy recommendations based on EasyOncology.
This will then provide the opportunity for a prospective cohort study, with clinics with decision-supported tumor boards and clinics without digital tumor board support serving as cohorts to be compared.

4. Inadequate details on the EasyOncology application and this could be improved in the main text

The content of EasyOncology was developed by experienced specialists from all relevant oncological specialties with the intention to provide evidence-based and unbiased first-line treatment recommendations for the most common cancer entities. The intuitive user interface and quality of the application led to a top 3 ranking in a worldwide comparison of 157 oncological applications in 2017. The content and therapeutic recommendations are based on expert-curated knowledge that is assimilated and interpreted from written and visual sources alike. More precisely, given oncological guidelines (i.e. S3-guidelines, NCCN), approval status of therapeutics and best-clinical practice of major cancer centers build the basis for EO's data platform. Query algorithms have been developed to effectively support the decision-making process, especially in complex cancer cases. EasyOncology was certified as a medical device in July 2020. To assure quality of given treatment recommendations, a continuous comparison with real-word treatment recommendations of various MTDs has been implemented. For this study, clinicopathological data of an uro-oncological MTD, namely the patient characteristics age, cancer stage, risk stratification, Gleason Score, and PSA-level (prostate specific antigen), were assessed to provide treatment recommendations using EasyOncology.

5. Funding source of this study- Needs to be explicit. How was this study funded?

The study was conducted as part of the authors' university research activities and did not receive separate funding. The EasyOncology app was developed and published by the company Easy Medical

Applications, which was founded as a university start-up by Dr. Thomas Elter. The authors involved in the evaluation are independent specialists and received no funding in cash or other assets.

If you have any questions, please do not hesitate to contact me.

With kind regards,

PD Dr. med. Thomas Elter

References

1. Bungartz, K.D., Lalowski, K. & Elkin, S.K. Making the right calls in precision oncology. *Nat Biotechnol* **36**, 692-696 (2018).
2. Atieh Graham, D.M. *et al.* Are treatment recommendations provided by cognitive computing supported by real world data (Watson for Oncology with Cota RWE) concordant with expert opinions? *Ann Oncol* **29 Suppl 8**, viii571 (2018).
3. Choi, Y.I. *et al.* Concordance Rate between Clinicians and Watson for Oncology among Patients with Advanced Gastric Cancer: Early, Real-World Experience in Korea. *Can J Gastroenterol Hepatol* **2019**, 8072928 (2019).
4. Jie, Z., Zhiying, Z. & Li, L. A meta-analysis of Watson for Oncology in clinical application. *Sci Rep* **11**, 5792 (2021).
5. Keikes, L. *et al.* The first steps in the evaluation of a "black-box" decision support tool: a protocol and feasibility study for the evaluation of Watson for Oncology. *J Clin Transl Res* **3**, 411-423 (2018).
6. Kim, E.J. *et al.* Early experience with Watson for oncology in Korean patients with colorectal cancer. *PLoS One* **14**, e0213640 (2019).
7. Kim, M.S. *et al.* Artificial intelligence and lung cancer treatment decision: agreement with recommendation of multidisciplinary tumor board. *Transl Lung Cancer Res* **9**, 507-514 (2020).
8. Lee, W.S. *et al.* Assessing Concordance With Watson for Oncology, a Cognitive Computing Decision Support System for Colon Cancer Treatment in Korea. *JCO Clin Cancer Inform* **2**, 1-8 (2018).
9. Mao, C. *et al.* Concordance Between Watson for Oncology and Multidisciplinary Teams in Colorectal Cancer: Prognostic Implications and Predicting Concordance. *Front Oncol* **10**, 595565 (2020).
10. Pan, H. *et al.* Concordance assessment of Watson for Oncology in breast cancer chemotherapy: first China experience. *Transl Cancer Res* **8**, 389-401 (2019).
11. Sampige Prasannakumar, S. *et al.* Double blind concordance study of colo-rectal cancer treatment recommendations between artificial intelligence advisory programme watson for oncology (WFO) & multidisciplinary tumor board (MDT). *Ann Oncol* **29 Suppl 8**, viii137 (2018).
12. Somashekhar, S.P. *et al.* Watson for Oncology and breast cancer treatment recommendations: agreement with an expert multidisciplinary tumor board. *Ann Oncol* **29**, 418-423 (2018).
13. Suwanvecho, S. *et al.* Comparison of an oncology clinical decision-support system's recommendations with actual treatment decisions. *J Am Med Inform Assoc* **28**, 832-838 (2021).
14. Yao, S., Wang, R., Qian, K. & Zhang, Y. Real world study for the concordance between IBM Watson for Oncology and clinical practice in advanced non-small cell lung cancer patients at a lung cancer center in China. *Thorac Cancer* **11**, 1265-1270 (2020).
15. You, H.S. *et al.* Concordance of Treatment Recommendations for Metastatic Non-Small-Cell Lung Cancer Between Watson for Oncology System and Medical Team. *Cancer Manag Res* **12**, 1947-1958 (2020).
16. Yu, S.H. *et al.* Early experience with Watson for Oncology: a clinical decision-support system for prostate cancer treatment recommendations. *World J Urol* **39**, 407-413 (2021).
17. Yun, H.J. *et al.* Adequacy and Effectiveness of Watson For Oncology in the Treatment of Thyroid Carcinoma. *Front Endocrinol (Lausanne)* **12**, 585364 (2021).

18. Zhang, W., Qi, S., Zhuo, J., Wen, S. & Fang, C. Concordance Study in Hepatectomy Recommendations Between Watson for Oncology and Clinical Practice for Patients with Hepatocellular Carcinoma in China. *World J Surg* (2020).
19. Zhao, X. *et al.* Concordance between treatment recommendations provided by IBM Watson for Oncology and a multidisciplinary tumor board for breast cancer in China. *Jpn J Clin Oncol* **50**, 852-858 (2020).
20. Zhou, N. *et al.* Concordance Study Between IBM Watson for Oncology and Clinical Practice for Patients with Cancer in China. *Oncologist* **24**, 812-819 (2019).
21. Zou, F.W., Tang, Y.F., Liu, C.Y., Ma, J.A. & Hu, C.H. Concordance Study Between IBM Watson for Oncology and Real Clinical Practice for Cervical Cancer Patients in China: A Retrospective Analysis. *Front Genet* **11**, 200 (2020).
