# Supplementary material for: Validation and implementation of a mobile app decision support system for prostate cancer to improve quality of tumor boards
Source: PLOS Digit Health. 2023 Jun 7;2(6):e0000054. doi: 10.1371/journal.pdig.0000054 (PMC10246794; doi:10.1371/journal.pdig.0000054)
Supplement: Attachment — Submitted filename: Response to Reviewers.docx [file pdig.0000054.s002.docx]

**Subject:	Rebuttal Letter**

Dear Dr. Hadjiat, Dear Oliver Ian Tolentino, Dear Reviewers and Editors,

Thank you very much for your critical review of our manuscript.

We would like to comment on the individual points of critic:

**Comments to the Author**

1. If the authors have adequately addressed your comments raised in a previous round of review and you feel that this manuscript is now acceptable for publication, you may indicate that here to bypass the “Comments to the Author” section, enter your conflict of interest statement in the “Confidential to Editor” section, and submit your "Accept" recommendation.

Reviewer #1: (No Response)

**Reviewer #2: All comments have been addressed**

--------------------

2. Does this manuscript meet PLOS Digital Health’s [publication criteria](https://journals.plos.org/digitalhealth/s/journal-information#loc-criteria-for-publication)? Is the manuscript technically sound, and do the data support the conclusions? The manuscript must describe methodologically and ethically rigorous research with conclusions that are appropriately drawn based on the data presented.

Reviewer #1: (No Response)

**Reviewer #2: Yes**

--------------------

3. Has the statistical analysis been performed appropriately and rigorously?

Reviewer #1: (No Response)

**Reviewer #2: N/A**

--------------------

4. Have the authors made all data underlying the findings in their manuscript fully available (please refer to the Data Availability Statement at the start of the manuscript PDF file)?

The [PLOS Data policy](https://journals.plos.org/digitalhealth/s/data-availability) requires authors to make all data underlying the findings described in their manuscript fully available without restriction, with rare exception. The data should be provided as part of the manuscript or its supporting information, or deposited to a public repository. For example, in addition to summary statistics, the data points behind means, medians and variance measures should be available. If there are restrictions on publicly sharing data—e.g. participant privacy or use of data from a third party—those must be specified.

Reviewer #1: (No Response)

**Reviewer #2: Yes**

--------------------

5. Is the manuscript presented in an intelligible fashion and written in standard English?

PLOS Digital Health does not copyedit accepted manuscripts, so the language in submitted articles must be clear, correct, and unambiguous. Any typographical or grammatical errors should be corrected at revision, so please note any specific errors here.

Reviewer #1: (No Response)

**Reviewer #2: Yes**

--------------------

6. Review Comments to the Author

Please use the space provided to explain your answers to the questions above. You may also include additional comments for the author, including concerns about dual publication, research ethics, or publication ethics. (Please upload your review as an attachment if it exceeds 20,000 characters)

Reviewer #1: I thank the authors for this manuscript draft, and especially the great work they are doing in developing EasyOncology DSS for oncology. I believe their approach in using DSS, in combination with / controlled by the expert clinicians is exactly the right method to approach as complex and heterogenous indication area as oncology with various cancers and subpopulations is. Authors’ high level objective in this work is in replacing the valuable time of MTB in assessing trivial cases with DSS based automation is also correct approach when moving stepwise from AI assisted humans to more fully automated decision making.

**I do have some comments and proposals on the latest article revision. I believe some of them are more critical before this manuscript is ready to be published.**

GENERAL COMMENTS
• **You correctly mention in the abstract and discussion, that cross-validation over multiple clinics is required to further increase decision quality and avoid potential clinic bias. This is exactly as it should be, however as the EO application and advice by DSS evaluated in this manuscript are based(?) on PCa treatment practises specifically in Germany, I am left to consider whether your DSS and cross-validation is then applicable to treatment practises in Germany only. At least EO is available in German only. I can only assume based on my personal experience that SoC varies a lot in different countries, especially so in the earlier stages on PCa. I believe this should be mentioned as a limitation, at least in the discussion, or if the CV is done over several countries, perhaps this should be then mentioned to tackle this challenge?**

🡪 The medical content of EasyOncology is based on German (S3) guidelines. Thus, treatment recommendations are primarily addressed to clinicians in German hospitals. Therefore, our premise was that SoC for prostate cancer varies between German hospitals. Hence, we postulate that precisely these hospitals need to be compared with respect to their treatment decisions in order to increase decision quality and to avoid clinical bias. Nonetheless, we included the following sentence in our discussion taking your valuable feedback into account; “Another limitation is certainly that the reference recommendations are based on the German S3 guidelines and thus the methods and results cannot be transferred to other countries uncritically.”

• **Authors use both MTD and MTB abbreviations for multidisciplinary tumor board. Please unify.**

🡪 Abbreviations were unified in the text.

• **In general, should the figure titles be also within the figures too?**

🡪 To the best of our knowledge, we formatted all figures according to PLOS formatting guidelines. Accordingly, we did “not include author names, article title, or figure number/title/caption within figure files.” ([https://journals.plos.org/digitalhealth/s/figures#loc-how-to-submit-figures-and-captions](https://journals.plos.org/digitalhealth/s/figures#loc-how-to-submit-figures-and-captions)). Regardless, we can change the formatting before publishing if requested?

• **The quality of figure images is also rather poor**

🡪 The quality of all Figures was adjusted/enhanced to fulfill the “Figure File Requirements“ by PLOS and their quality was checked through the PACE software as recommended. The quality of the figures in the pdf created is indeed poor thus the original tifs are better and as shown in the test by PACE fine for publishing.

**• Check the font for different header levels in the manuscript for ease readability.**

🡪 In line with PLOS formatting guidelines, we used “Level 1 heading for all major sections (Abstract, Introduction, Materials and methods, Results, Discussion, etc.) or bold type, 18pt font.” Level 2 headings were used “for sub-sections of major sections” or “bold type, 16pt font”. (<https://storage.googleapis.com/plos-published-prod/9cba/PLOS%20Manuscript%20Body%20Formatting%20Guidelines.pdf?X-Goog-Algorithm=GOOG4-RSA-SHA256&X-Goog-Credential=wombat-sa%40plos-prod.iam.gserviceaccount.com%2F20221121%2Fauto%2Fstorage%2Fgoog4_request&X-Goog-Date=20221121T122727Z&X-Goog-Expires=86400&X-Goog-SignedHeaders=host&X-Goog-Signature=3447268d075d50779647b36831a1fe32ce14fd5fed444f9eac1da8055b17833521c873174d6481465cabdd8a554b26059852a620fa3ded6a4adbaa307027be4570fbb348d30b7a5f49f389ab4fa3ed16d7a2442f6be7ac734ccc69cc5ca68f34baea85ed4913a4946f058aac79f8551681930a49942b684704db47324e829e102b828a9e95910127c8fd59a79a80bf81ca51a8ebd798a2eabb3e50de6e30df2d47a00b73ccf34530e796817e1e21c2da5938414b90ffa37c381d8d919e551d68ed207159e0d1efa9813abbedf73892efda456b0230afee1cd6b6db6242e1f08553c06accb91d34dd7d007c43766170e5f98fc61eb121385a5a49bba35a43bf01>)

**• Overall, the references in the text to table 1, figure 3 and figure 4 do not seem to be perfect in relation to their aimed location within the text.**

🡪 We corrected the manuscript for mismatches in reference allocations.

**• Use of references, square brackets vary through the manuscript a bit, at least in discussion. Sometimes after the period, sometimes before. Please unify.**

🡪 Reference citations were unified in accordance with PLOS guidelines (“[1]” or “[2-5]” or “[3,7,9]).

Table 1:
**• Some values contain size of N/A population, in some cases such as clinical stage & stratification the size of not mentioned.**

🡪 The information on clinical stage and stratification was not taken directly from the tumour board mask, but was derived from the information in the tumour board registration prose text and the direct variables from the registration mask. The clinical stage and stratification could thus be specified without gaps.

**• What is the “a” in superscript in the footnote?**

🡪 Was deleted.

Figure 1:
**• please check the language of figure title**

🡪 The figure title was changed from “Flow chart patient cohort with prostate carcinoma” to “Flow chart of patient case selection process.”

Figure 4:
**• please elaborate the title**

🡪 The title was changed/elaborated from “Concordance rates by Tumor Stage” to “Treatment concordance rates between a multidisciplinary tumor board and the application EasyOncology according to prostate cancer tumor stage”.

ABSTRACT
**• In the author summary you write “Unfortunately, current digital tools that have been developed to support clinicians on the process of decision-making, have failed to provide treatment recommendations with sufficient accuracy” – now this is a rather bold and should perhaps be softened by using “having difficulties” or similar, instead of “failing”.**

🡪 The sentence was changed/softened as suggested to “Unfortunately, current digital tools that have been developed to support clinicians on the process of decision-making, have difficulties to provide treatment recommendations with sufficient accuracy”.

**• You also speak here about newly diagnosed patients here but do not mention anything about this in the study design and patients? Was this an inclusion criteria for retrospective analysis, and if not, are the results applicable for patients with longer patient journey? There seems to something in the discussion about this but I believe it should belong to design and patients, as well.**

🡪 Yes, “newly diagnosed patients” was an inclusion criteria. The manuscript section “Study Design and Patients” was adjusted accordingly. In particular the sentence “Data sets of 2412 prostate cancer cases presented at university uro-oncology MTD during the time period of 2014-2018 were screened for inclusion criteria.” was changed to “Data sets of 2412 newly diagnosed prostate cancer patient cases cases presented at university uro-oncology MTDB during the time period of 2014-2018 were screened for inclusion criteria.

INTRODUCTION
**• In the end of introduction I would suggest a small change: “The aim of this clinical research is to implement the aforementioned technology for validation and quality assurance of a urological tumor board in NAME OF HOSPITAL”.**

🡪 The sentence was changed as suggested to: “The aim of this clinical research is to implement the aforementioned technology for validation and quality assurance of a urological tumor board at the University Hospital of Cologne.”

MATERIAL AND METHODS
Smartphone application
**• I do believe the authors that EO has been adequately developed, and credible, but the manuscript itself is still in my opinion failing to answer to original review critique #4: what is it that EO does? The reader would need to download the application (not available in the country where I live). What are the possible treatment recommendations (input => OUTPUT)? I can understand what the input is, but I don’t understand what the output from DSS is here? It would increase the contextual understanding of these results a lot.**

🡪 We adjusted the manuscript taking into account your valuable feedback: “Treatment recommendations for a given cancer entity are narrowed down by EO according to a specific query of clinicopathological factors. In less complex tumor entities, this may only be a question of staging, whereas more complex tumors and treatment situations require the input of further info, e.g., molecular pathology or age to reach a sufficiently granular recommendation. There are two main formats in which EO delivers this information. Either in condensed, but continuous texts, where treatment recommendations for an entire stage of a cancer entity are summarized, or in the form of a very short and specific recommendation for a narrow constellation of entity, stage and additional factors”

**• What is the medical device classification of EO?**

🡪 EasyOncology was certified as a class I medical device in July 2020 according to MDD rule 1 and 12 (we adjusted the manuscript accordingly).

Study design and patients
• **How is the second round of analysis relevant for these results or this manuscript?**

🡪 We first categorize response pairs to lay the groundwork for in depth analysis of EO’s query. In particular, in the first round of analyses, EO’s treatment recommendations were grouped into one of four categories similar to the evaluation concept of previous publications on Watson for Oncology by IBM. The second review was used to analyse the non-concordant cases in order to improve EO’s algorithm by identifying potential errors in its query.

DATA ANALYSIS AND RESULTS
**• MS Excel version, if it really has been used for data analysis?**

🡪 MS Excel Version 16 was used in order to calculate concordance rates and to illustrate Fig 3 and Fig 4 (we added the version in the manuscript).

DISCUSSION
**2nd paragraph: I would change the questions into statements that are then backed by references.**

🡪In this particular case we prefer to formulate the sentences as questions since we believe that they have not been answered adequately by academia.

**• You are writing that “all attempts of AI-systems to reliably provide even standard therapeutic recommendations for first-line therapy have been disappointing”. I wouldn’t use such a strong statement with one example.**

🡪 We changed/softened the sentence and used the word “most” instead of “all” (in the revised version of the manuscript).

**• Finally, I would touch shortly the use case vs reliability here – is it better that system over-recommends treatments as what are the consequences when someone who needs to be treated won’t get it? On the other hand, safety issues are waiting if over-recommending treatments.**

🡪 Since our algorithm is mainly based on S-3 guidelines, cases where EO gives over-recommendations or under-recommendations shall hopefully not exist or be limited to a very few cases. In the light of this argument we believe that the proposed adjustment will not enhance our discussion.

Reviewer #2: (No Response)

--------------------

7. PLOS authors have the option to publish the peer review history of their article ([what does this mean?](https://journals.plos.org/digitalhealth/s/editorial-and-peer-review-process#loc-peer-review-history)). If published, this will include your full peer review and any attached files.


**Do you want your identity to be public for this peer review?** If you choose “no”, your identity will remain anonymous but your review may still be made public.

For information about this choice, including consent withdrawal, please see our [Privacy Policy](https://www.plos.org/privacy-policy).

Reviewer #1: No

Reviewer #2: Yes: Yacine HADJIAT

--------------------

[NOTE: If reviewer comments were submitted as an attachment file, they will be attached to this email and accessible via the submission site. Please log into your account, locate the manuscript record, and check for the action link "View Attachments". If this link does not appear, there are no attachment files.]

**While revising your submission, please upload your figure files to the Preflight Analysis and Conversion Engine (PACE) digital diagnostic tool, https://pacev2.apexcovantage.com/. PACE helps ensure that figures meet PLOS requirements. To use PACE, you must first register as a user. Registration is free. Then, login and navigate to the UPLOAD tab, where you will find detailed instructions on how to use the tool. If you encounter any issues or have any questions when using PACE, please email PLOS at figures@plos.org. Please note that Supporting Information files do not need this step.**

🡪 We used the proposed conversion engine PACE for our figures and uploaded the files as suggested.

__________________________________________________
In compliance with data protection regulations, you may request that we remove your personal registration details at any time. (Use the following URL: https://www.editorialmanager.com/pdig/login.asp?a=r). Please contact the publication office if you have any questions.

If you have any questions, please do not hesitate to contact me.

With kind regards,

PD Dr. med. Thomas Elter
