# Supplementary material for: Validation and implementation of a mobile app decision support system for prostate cancer to improve quality of tumor boards
Source: PLOS Digit Health. 2023 Jun 7;2(6):e0000054. doi: 10.1371/journal.pdig.0000054 (PMC10246794; doi:10.1371/journal.pdig.0000054)
Supplement: Attachment — Submitted filename: 230331Response to reviewers.docx [file pdig.0000054.s003.docx]

**Subject:	Response to the editor**

Dear Haleh Ayatollahi, dear Oliver Ian Tolentino, dear Dr. Padmanesan Narasimhan, dear Reviewers and Editors,

Thank you very much for your critical review of our manuscript.

We would like to comment on the individual points of critic:

**Editor comment #1:**
*„The title seems too long. Please make it shorter. The authors can remove the second part of the title starting with “Analyzing…”.“*

**Author changes:**
Validation and implementation of a mobile app decision support system for prostate cancer to improve quality of tumor boards

**Editor comment #2**
*„Please choose the keywords based on the MeSH terms, as well.“*

**Author changes:**
MeSH terms: decision support system, DSS, digital decision support, tumor board, MTD, prostate cancer

**Editor comment #3**
*„Please follow the journal instructions to provide a concise unstructured abstract.“*

**Author changes:**
Now, the abstract is conceptually divided into three sections (Background/Objective, Methodology/ Principal Findings, and Conclusions/Significance) without distinct headings within the article file

**Editor comment #4**

„In the introduction, although the authors reviewed the existing literature, they need to explain the gap in the existing knowledge, too.“

**Author changes:**

An important reason for the poor performance of AI systems is the lack of high-quality training datasets. Masses of normalized training data are available for AI image recognition systems, but not for AI applications that are intended to model regular oncology care.

In addition to the lack of properly organized and validated training data, another problem is the limited resource of experts initially required for human interpretation and evaluation of AI results.

At the end of the day, then, a machine learning tool can only be as good as the data available for training and the trainers who evaluate the AI results.

**Editor comment #5**

*„In the methods section, please explain the study design and participants first and then explain the design of the smartphone application.“*

**Author changes:**

The section „Smartphone application“ now follows the section „Study design and patients“

**Editor comment #6**

*„In page 5, the authors noted “The content of EasyOncology was developed by experienced specialists…”. It is important to elaborate on this part and make it clear how and using which methodology the content was developed.“*

**Author changes:**

The content of EasyOncology was created by experienced clinical specialists from different cancer centers and oncology practices. Diagnostic and therapeutic recommendations follow the usual evidence-based guidelines of the professional societies, best clinical practice and the approval status of oncological therapeutics.

Medical editors revised new content, discussed conflicting information with the authors, and assured EO updates in predetermined time intervals. Quality of decision algorithms was assured by implementing a constant comparison with real world decisions given in tumor boards.

**Editor comment #7**

*„In the methods section, please add the inclusion and exclusion criteria for the patients.“*

**Author changes:**
Inclusion criteria for the study were prostate cancer cases for which a therapy recommendation was given in the uro-oncological tumor board of the University Hospital in the period from 2014 to 2018. Data sets of 2412 patient cases included then and screened for exclusion criteria: 140 case discussions not addressing therapeutic procedures, such as specific questions about histopathology or how to obtain a biopsy were excluded, as well as another 264 cases without therapeutic decisions due to pending clinical information. Another 135 cases recommended for clinical trials (n=50) or complex cases with more than one active tumor entity (n=85) were also excluded (Fig 1).

**Editor comment #8**

*„The readers may need to know more about the technical aspects of the CDSS. Please add some figures of the system and provide an example (flow chart) to show how the system worked. Moreover, please make sure that the figures are visible in the submitted file.“*

**Author changes:**

As depicted in Fig. 3 EO requests clinicopathologic patient data to generate treatment recommendations in a stepwise fashion. The number of clinicopathologic variables necessary to generate treatment recommendations depends on the complexity of the patient case.

For example in case of a “localized PC” only two clinicopathologic variables (i.e. risk group and histology) are required by EO to give a treatment recommendation. In more complex cases (i.e. nmCRCP) three clinicopathologic variables are requested by EO to provide a treatment recommendation.

**Fig 3**. **Query algorithm.**
The relevant information is requested by EO’s query algorithm depending on the selected initial clinical status. Abbreviations:
TNM: Classification of Malignant Tumors; ECOG: Eastern Cooperative Oncology Group; PSA: Prostate-Specific Antigen; ISUP: International Society of Urological Pathology; PC: prostate cancer; mHSPC: metastatic Hormone-Sensitive Prostate Cancer; nmHSPC: non-metastatic Hormone-Sensitive Prostate Cancer; nmCRPC: non-metastatic Castration-Resistant Prostate Cancer; mCRPC: metastatic Castration-Resistant Prostate Cancer; TUR-P: Transurethral Resection of the Prostate; RPE: Radical Prostatectomy

**Editor comment #9**

„The evaluation methods need to be explained in detail. The authors could also evaluate the specificity, sensitivity, accuracy, etc.“

**Author changes:**

From a statistical point of view, the determination of sensitivity and specificity is not applicable, since a “gold standard” for correct treatment decisions does not always exist. In other words, cases can be non-concordant since EO strictly adheres to society guidelines whereas decisions from MDT might deviate from standard for individual reasons. The idea to calculate a sensitivity does impose a mutually exclusive argument where only one party (EO or the MDT) is correct.

**Editor comment #10**

„In Table 1, some figures are the same and their percentages are different and vice versa. Please re-check Table 1.“

**Author comment:**
“In the original version, the absolute (n=) and relative (%) values referred to the total number of subgroups indicated in the header of each column. At first glance, this led to seemingly inconsistent results.

In the revised manuscript, all figures refer to the total number of cases studied.”

**Author changes:**

Original version

| ***Characteristic*** | ***Total,***  ***n=1873*** | ***Concordant group,***  ***n=1856*** | ***Discordant Group,***  ***n=17*** | ***P Value*** |
| --- | --- | --- | --- | --- |

Revised version

| ***Characteristic*** | ***Total,***  ***n=1873*** | ***Concordant group,*** | ***Discordant Group,*** | ***P Value*** |
| --- | --- | --- | --- | --- |

**Editor comment #11**

*„The possible reasons for reaching concordance rates of (100%) need to be explained in the discussion section.“*

**Author changes:**

The agreement of 100%, achieved in our analysis in stage III, indicates only the simpler decision for active therapy in this risk constellation compared with the early stages of prostate cancer.

**Editor comment #12**

*“We need to see a conclusion section after the discussion”*

**Author changes:**

Our study showed a high concordance rate (99%) between the treatment recommendations provided by EO and treatment recommendations provided by MTD for patients with prostate cancer. Concordance rates were consistent across different age groups, stages of disease, and risk profiles. Multivariate logistic regression analysis did not reveal any significant variables associated with a decrease in concordance rates. The Gleason score and the prognostic Grade (ISUP) were univariat significantly associated with concordance rate. The analysis of discordant cases can be used to further optimize recommendations provided by EO. Overall, our results suggest that EO is a promising tool to assist clinicians in providing reliable treatment recommendations for prostate cancer patients.

If you have any questions, please do not hesitate to contact me.

With kind regards,

Yasemin Ural
